# Productional data of primiparous dairy cows reared in different social environments during the first 8 weeks after birth

**DOI:** 10.1016/j.dib.2022.108273

**Published:** 2022-05-16

**Authors:** Barbora Valníčková, Radka Šárová, Ilona Stěhulová

**Affiliations:** a Department of Ethology, Institute of Animal Sciences, Prague 104 00, Czech Republic; b Department of Ethology and Companion Animal Science, Faculty of Agrobiology Food and Natural Resources, Czech University of Life Sciences in Prague, Prague 165 21, Czech Republic

**Keywords:** Dairy Cattle, Group housing, Rearing conditions, Milk production, Activity, Body weight, Longevity, Performance

## Abstract

This paper is composed of 5 datasets describing primiparous milk production, reproduction, body weight, activity and whole life longevity and reproductional data in dairy cows that had been reared either with or without mother for the first four days after birth and either in single housing or housing in groups of four between 1 and 8 weeks of age. The datasets contain the following variables- survival to the first lactation, date of first successful insemination, milk parameters per day (such as sum of milk yield, milk electrical conductivity and milking time), activity and body weight, all these collected during the first standardized lactation of 305 days. Cows’ longevity, reproduction and other management events were recorded during the whole life of experimental animals (such as inseminations, pregnancy diagnostics, group changes etc.). Calves’ body weight was measured first 12 weeks of life of the experimental animals. The data include the information about the type of housing (with or without mother, individual vs group housing) in the early ontogeny period and two different breeds (Holstein and Czech Fleckvieh). Data on the milk parameters, body weight and activity were collected twice a day by commercially used precision dairy monitoring technologies. Data on survival to the first lactation, longevity, first successful insemination and other events were recorded by farm managers on farm basis. Data on body weight of animals during early ontogeny were taken after birth, at 4 d of age, at 7 d of age, and then weekly until 12 weeks of age. The data can be used for further analyses of the influence of parameters from early ontogeny on cow performance, especially during the first lactation. This information can be useful for researchers and other stakeholders investigating the influence of early ontogenetic social environment on the dairy cattle performance and welfare.

## Specifications Table

**Table:** 

Subject	Animal Science and Zoology
Specific subject area	Dairy cattle management and production
Type of data	Table
How the data were acquired	Electronic scale: Eziweigh2, Tru-Test Ltd., Auckland, New Zealand- calf weight (kg) Commercially used precision dairy monitoring technologies measuring physiological, behavioral, and production variables for individual animals: Dairy management systems made and produced by company Afimilk Ltd. Programmable milking point controller: AfiMilk MPC™ milk meter- milk yield (g), milk electrical conductivity (mmHO), milking time (sec) Accelerometer sensors: Afitag pedometers on left hind leg- activity (e = number of steps) Weighting system: automatic walk- over weighting system AfiWeight- weight of dairy cows (kg) Anntenas: IDeal Afi identification systém, the identification system's sensors and active identification sequence in Afi systems-Transfer of data from sensors to AfiFarm™ software Veterinary and management records: written by hand of farm manager to the Afifarm™ 4.1 [1] software- All measurements in „Events“ dataset (see Tab. 2) All data were taken within the daily routines of commercial dairy farm Software: Afifarm™ 4.1 [1]- Data collection SAS 9.4 [2]- Data filtration
Data format	Raw Filtered
Description of data collection	Calves were randomly allocated to treatments “Mother” and “Housing”. They were weighted 3 times in first week and then weekly for 12 weeks. During lactation data were collected after milking by precision dairy monitoring technologies. For numerical variables, one final number for each variable was given per day. Other data were collected manually. For description of variables see Tables 1 and 2.
Data source location	Institution: Institute of Animal Sciences City: Prague Country: Czech Republic Latitude and longitude (and GPS coordinates) for collected data: 50°02’19.7”N 14°36’36.0”E
Data accessibility	Repository name: Mendeley Data Data identification number: 10.17632/j42n4x723g.2 Direct URL to data: https://data.mendeley.com/datasets/j42n4x723g/2
Related research article	B. Valníčková, I. Stěhulová, R. Šárová, M. Špinka, The effect of age at separation from the dam and presence of social companions on play behavior and weight gain in dairy calves, Journal of Dairy Science, 98 (2015) 5545-5556 [3]. https://doi.org/10.3168/jds.2014-9109 B. Valníčková, R. Šárová, M. Špinka, Early social experiences do not affect first lactation production traits, longevity or locomotion reaction to group change in female dairy cattle, Applied Animal Behaviour Science, 230 (2020) 105015 [4]. https://doi.org/10.1016/j.applanim.2020.105015

## Value of the Data

• This dataset shows association of different types of housing during early ontogeny in calves and milk production, body weight (in early ontogeny and in first lactation) and activity in dairy cows during their first lactation in two different breeds.
• These data can be beneficiary for researchers and other stakeholders working in cattle welfare improvement.
• These data can be further used for analysis or metanalysis of cow's production with connection to early ontogeny rearing and welfare.
• High quality data are possible to provide by commonly used commercial precision dairy monitoring system.
• These data can be further used for dairy cattle welfare improvement
• These data can be further used for assessment of welfare indicators in dairy systems.

## 1 Data Description

We submit five data sets, which were recorded by commercially used Precision dairy monitoring technologies measuring physiological, behavioral, and production variables for individual animals. For closer description of all variables in all datasets please see the Tables 1 and 2.

**Table 1:** Description of individual variables in all datasets.

Name of the variable	Description of variable	Dataset name
CowID CalfID	ID -registration number of the individual animal	­ Survival ­ Service period ­ Milk yield ­ Daily data ­ Calfweight
Mother	When calf was separated from the mother 0 - during 24 h post partum 4 - 4th day post partum	­ Survival ­ Service period ­ Milk yield ­ Calfweight
Housing	Housing of the calves after separation from mother until 8 weeks of age I – individually G - groups of 4 calves	­ Survival ­ Service period ­ Milk yield ­ Calfweight
Breed	H - Holsein cattle C - Czech Fleckvieh cattle	­ Survival ­ Service period ­ Milk yield ­ Calfweight
Date	Date of the measurement	­ Daily data
1st lactation survival	Did the heifer survive until the start of the first lactation? YES - the heifer started her first lactation in the herd NO - the heifer died or was culled before first calving	- Survival
Exit age	Age in days when animal was culled	- Survival
Age pregnancy	Age in days when heifer was successfully inseminated (and later confirmed as pregnant).	­ Service period
Sum of milk	Total milk yield in grams for standardized lactation (305 days).	­ Milk yield
Group	Number of the group where animal is placed on this date	­ Daily data
Yield(gr)	Total daily milk yield in grams	­ Daily data
Conductivity	Maximal electrical conductivity of the milk measured given day in (mmHO)	­ Daily data
Milking_Time(Sec)	Milking session in seconds, higher number from two daily milkings was reported	­ Daily data
Body Weight(kg)	Average weight of the animal in kilograms per day	­ Daily data
Activity(steps/hour)	Number of steps per hour- Highest value measured this day was reported	­ Daily data
Birth 4days 1weeks-12weeks	Body weight in kilograms weighted in birth, 4 days, 1st -12th week of life of experimental animal	­ calfweight

**Table 2:** Description of individual events.

Name of the event	Description of the event
Abortion	Natural loss of an embryo or fetus before it is able to survive independently.
Birth	Birth of the focal animal
Calving	Give birth to a calf
Dry	Ending milk production period of the cow before the next calving or exit from the herd
Entry	Entry animal to the herd due to birth or purchase
Exit	Exit animal from the herd due to death or sale
Heat	Female sexual receptivity in oestrus
Change Group	Move of the animal from one group to another
Insemination	Introduction of sperm into a female's cervix for the purpose of achieving a pregnancy
Not for Insemination	Animal is defined by the herd manager not for insemination
OK Not Pregnant	Animal confirmed not pregnant after examination
PD(+)	Animal confirmed pregnat after examination

The first data set is called “Survival“, and it shows the experimental treatment of animals, age when experimental animals were culled and whether they survived until their first lactation.

The second data set is named “Service period” and shows the experimental treatment of animals and the date of their first successful insemination. The insemination was considered successful if it was later confirmed as a successful pregnancy.

The third dataset is called “Daily data” and shows daily data of primiparous cows about milk yield, milking time, milk electrical conductivity, body weight and activity of every experimental animal until the end of the standardized lactation (305 days) or death of the animal if it occurred before the end of the first lactation.

The fourth dataset is called “Events” and shows a selection of important veterinary or management events and procedures which experimental animals underwent during their whole life from birth until their exit from the herd (culling or death).

The fifth dataset is called “Calf weight” and shows the body weight of the experimental animals when they were born, at 4th day, 1st week and then every week until 12th week of their life.

Filtered datasets are Survival, Service period and Milk yield which were formed with the use of Daily data and Events datasets. Daily data, Events and Calfweight are raw data.

## 2 Experimental Design, Materials and Methods

### 2.1 Animals and Experimental Procedures

All observations were realized at the experimental farm of the Institute of Animal Science in Prague, Czech Republic. In the experiment, 40 female calves of two breeds of domestic cattle (11 Czech Fleckvieh and 29 Holstein) were used, corresponding to the composition of the dairy farm herd. Data about experimental animals were collected from their birth until their death/ exit from the farm. Experimental heifer calves were randomly allocated, at the time of their birth, to one of four treatments according to a two factorial design, which gave 4 treatments in total: 0 days with mother x 4 days with mother and after single housing x group housing.

The first treatment differentiated between those calves kept with and those without their *mother* during the first 4 days after parturition. The calves were kept with their mothers in a straw-bedded calving pen (4 × 3 m) until separation.After their separation from the mother all calves were housed individually until 1 week of age, to ensure that they knew how to drink milk from an open bucket.

A *companion* treatment was applied thereafter, which distinguished between calves reared with 3 other social companions in *group* pens and calves reared in *individual* pens between 1st and 8th weeks of age. The individual pen composed of an individual plastic hutch (1.2 × 1.4 m, 1.7 m^2^) and an outside run (1 × 1.2 m, 1.2 m^2^). The calves in individual pens had visual but not tactile contact with two other calves in the neighbouring pens, as a gap of 40 cm was left between them. The *group* pen consisted of 4 individual plastic hutches (4 × 1.7 m^2^) connected by the central outside run (4 × 1.2 m^2^). All hutches had solid concrete floor, and were richly bedded with straw (4 kg of straw/calf, huts were cleaned and added new straw 3 times per week). The outside run had solid floor made of concrete, that was cleaned once a week. After the separation of calves from their mother, calves were fed 2 L of milk (or colostrum before 5 days of age) twice a day from open buckets until 8 weeks of age. All calves had ad libitum access to hay, starter mixture and water during the entire rearing period.

In the *group housing*, 2 heifer calves and 2 bull calves of similar age were housed together in each of 20 groups, but only 1 heifer in each group was monitored as the experimental animal. Thus, we observed 40 experimental animals in total, 10 experimental animals per each treatment. The calves were randomly assigned to the 4 treatments. The breed of the calves and the sex could not be fully randomized across the treatments due to the farm conditions. The *group housing* was moderately balanced for breed at the beginning of the experiment (Holstein to Czech Fleckvieh ratio was 16:4 in the *individual housing* treatment and 13:7 in the *group housing* treatment) and nearly balanced at 12 weeks of age (14:4 individual housing, 12:4 group housing). The treatment of calves kept first 4 days after birth with or without mother was not balanced in terms of breed neither at the beginning (the breed ratio being 17:3 in calves immediately separated from mother and 12:8 in calves kept with mothers) nor at the end of the experiment at 12 weeks (the breed ratios being 15:1 and 11:7, respectively).

At 8th week of age, all calves were weaned abruptly from the milk and moved at the same time to a large group pens. After weaningthey had ad libitum access to a starter mixture, hay and water. Calves coming from the *individual housing* treatment were grouped with 3 unfamiliar calves (1 female calf and 2 male calves). Calves in the *group housing* treatment were moved to a new group pen together with their penmates and therefore their social environment did not change. In each of the groups, only 1 female calf was a focal animal. Calves were kept in these large group pens until 12th week of age. Large group pens consisted of a plastic hut (9 m^2^) and an outside run (9 m^2^). All areas had a solid concrete floor, and the hut was heavily bedded with straw (16 kg of straw, huts were cleaned and added new straw 3 times per week).

Starting at 12 weeks of age, all experimental animals became part of the production herd and they were submitted to the routine farm management together with all other female cattle on the farm.

After 12 weeks of age, the calves were moved to groups of 24 heifers where they were later inseminated (between 13 and 18 months of age) and stayed there until late gestation of their first pregnancy (5 month of pregnancy). The age of insemination was dependent on individual body weight and body condition.

At 5 months of pregnancy (late gestation period) the heifers were moved to the group of dry cows where they stayed until calving.

Animals in all mentioned groups were housed freely on solid concrete floor without bedding and with cubicles bedded with straw provided for lying. Number of cubicles was equal to number of animals in the group. Heifers, had access to an outside run with solid concrete floor. Heifers and pregnant heifers in groups were fed once a day with a Total mixed ratio (TMR) based on corn silage, haylage and straw. After entering the group of dry cows, late-gestation heifers were fed once a day with TMR based on haylage, straw and mineral additives. Scattered feed (TMR) was pushed back to the feeding bunk 4 times a day in all groups of heifers, pregnant heifers and dry cows. Before every delivery of fresh feed, old remaining feed was removed if any left.

Just before calving, the heifers in late gestation period were isolated and placed in a straw bedded calving pen. Within 24 h after calving the newly primiparous cows were moved to the group of about 50 lactating cows (GROUP 1). After the peak of lactation, when their milk yield dropped below 24 kg milk/ day, the cows were moved to the next group of lactating cows (GROUP 2) of about 48 animals. When their milk yield dropped further, the cows were moved to the last group of lactating cows (GROUP 3) consisting of approximately 60 animals. Two months before the next calving the cows were dried off and were moved to the group of dry cows (GROUP 4), where they weren't milked until the calving and start thenext lactation.

Lactating cows in all groups were housed freely and all these groups had solid concrete floor without bedding and cubicles bedded with straw. Cows in all groups had access to an outside run with solid concrete floor and cubicles bedded with straw placed under the roof (1 cubicle per cow). Manure from group pens was removed two times per day and straw in cubicles was changed once a week. Lactating cows were fed twice a day with TMR based on corn silage, haylage and straw with mineral and vitamin additives. Scattered TMR was pushed back to the feeding bunk 9 times per day. Before every delivery of fresh feed, all remaining feed was removed. Lactating cows were milked twice a day at 4am and 4pm in a tandem parlour with 3 × 3 stalls.

### 2.2 Data Collection

The calves were weighed with an electronic scale (Eziweigh2, Tru-Test Ltd., Auckland, New Zealand) immediately after birth, at 4 d of age, at 7 d of age, and then weekly until 12 weeks of age.

Data about milking, body weight and activity were automatically collected and processed by commercial Precision dairy monitoring systems created and produced by company Afimilk Ltd. Sensors of these systems have been developed with the capability to measure variables for individual cows [5] including milk yield, lying time, milk electrical conductivity [6], number of steps [7], and body weight [8]. Data about milk yield, electrical conductivity of milk and milking duration were measured during every milking by sensors of AfiMilk MPC™ milk meter placed in each milking stall. To get reliable information about electrical conductivity of milk during one milking, each 200 cubic centimetres (cc) of milk is checked while the milk flows through the milk meter [9]. Data about activity were collected through Accelerometer sensors Afitag pedometers placed on left hind legs of all cows.

Each cow was weighed on an automatic walk-over weighing system called AfiWeight, which automatically measures the cow's body weight and saves it in AfiFarm Software[1] database The system consists of a weighing platform installed en-route from the milking parlour, weighing the cows returning from every milking regularly.

A specific Pedometer ID number was assigned to each cow in the AfiFarm software [1] on the cow's card. The pedometer was used for the identification of the cow at the milking parlour and the AfiWeight station, where all measurements of milk production, activity and weight were taken. Data from Afitag pedometers and AfiMilk MPC™ milk meters were automatically collected during every milking, while data from AfiWeight were collected after every milking, when the cow walked over the weighing platform. Immediately after every milking and weighing, the data for each cow were scanned and sent together with the ID number to the central computer via IDeal Afi radiofrequency antenna. The data were then processed and stored by the dairy farm management software AfiFarm™ version 4.1 [1].

The highest number of steps per hour, the weight of milk, the body weight and the highest values of electrical conductivity and milking duration are noted in the AfiFarm™ software after everymilking and weighing sessions. The final numbers of daily data are given by the software after the last milking and weighing session each day. Information about calving, inseminations, pregnancy, transfer of an animal between the production groups and exit of the animal from the farm were added to the software manually by the manager of the farm according to the routine.

Filtered datasets were processed with software SAS 9.4 [2].

## Ethics Statements

In the experiment participated 40 females of domestic cattle. The experiment complied with the ARRIVE guidelines and was designed and performed in accordance with EU Directive 2010/63/EU for animal experiments and Czech law 246/1992 Coll. for the protection of animals against cruelty. The protocol was approved by the Institutional Animal Care and Use Committee of the Institute of Animal Science (No. 18/2004).

## CRediT authorship contribution statement

**Barbora Valníčková:** Conceptualization, Methodology, Validation, Investigation, Writing – original draft, Writing – review & editing, Visualization, Data curation. **Radka Šárová:** Conceptualization, Methodology, Validation, Formal analysis, Writing – original draft, Writing – review & editing, Supervision, Data curation. **Ilona Stěhulová:** Conceptualization, Methodology, Validation, Investigation, Resources, Writing – review & editing, Data curation, Supervision, Project administration.

## Declaration of Competing Interest

The authors declare that they have no known competing financial interests or personal relationships that could have appeared to influence the work reported in this paper.

## Data Availability

Productional data of primiparous dairy cows reared in different social environments during the first 8 weeks after birth (Original data) (Mendeley Data).
